# Electro-Conductive Modification of Polyvinylidene Fluoride Membrane for Electrified Wastewater Treatment: Optimization and Antifouling Performance

**DOI:** 10.3390/membranes15010001

**Published:** 2024-12-24

**Authors:** Jinzhuo Shi, Yisong Hu, Songhua Li, Wenqian Xiao, Yuan Yang, Jiayuan Ji

**Affiliations:** 1 Shaanxi Key Laboratory of Environmental Engineering, Xi’an University of Architecture and Technology, Xi’an 710055, China; shijinzhuo@xauat.edu.cn (J.S.); lisonghua@xauat.edu.cn (S.L.); wenqianxiao@xauat.edu.cn (W.X.); yuanyang@xauat.edu.cn (Y.Y.); 2 Key Laboratory of Northwest Water Resource, Environment and Ecology, Ministry of Education, Xi’an University of Architecture and Technology, Xi’an 710055, China; 3 Institute for Future Initiatives, The University of Tokyo, 7-3-1 Hongo, Bunkyo-ku, Tokyo 113-8654, Japan

**Keywords:** membrane bioreactor, response surface methodology, in situ oxidative polymerization, polypyrrole, antifouling

## Abstract

Electro-conductive membranes coupled with a low-voltage electric field can enhance pollutant removal and mitigate membrane fouling, demonstrating significant potential for electrified wastewater treatment. However, efficient fabrication of conductive membranes poses challenges. An in situ oxidative polymerization approach was applied to prepare PVDF-based conductive membranes (PVDF-CMs) and response surface methodology (RSM) was adopted to optimize modification conditions enhancing membrane performance. The anti-fouling property of the conductive membranes was analyzed using model pollutants. The results indicate that when the concentrations of the pyrrole, BVIMBF_4_, and FeCl_3_·6H_2_O are 0.9 mol/L, 4.8 mmol, and 0.8 mol/L, respectively, the electrical resistance of the PVDF-CM is 93 Ω/sq with the water contact angle of 31°, demonstrating good conductivity and hydrophilicity. Batch membrane filtration experiments coupled with negative voltage indicated that when an external voltage of 2.0 V is applied, membrane fouling rates for the conductive membrane filtering BSA and SA solutions are reduced by 17.7% and 17.2%, respectively, compared to the control (0 V). When an external voltage of 0.5 V is applied, the membrane fouling rate for the conductive membrane filtering HA solution is reduced by 72.6%. This study provides a valuable reference for the efficient preparation of conductive membranes for cost-effective wastewater treatment.

## 1. Introduction

Membrane separation technology has several advantages of high-quality effluent production, small footprint, and low energy consumption, making it an essential option in water/wastewater treatment [1,2]. Nowadays, pressure-driven membrane processes, such as microfiltration (MF), ultrafiltration (UF), nanofiltration (NF), and reverse osmosis (RO), have been widely utilized in drinking water purification and wastewater treatment [3,4]. Among such membrane-based processes, MF and UF are widely used in wastewater treatment because of their high membrane surface porosity and permeability. However, challenges still exist in the application of membrane technologies, such as membrane fouling and low selectivity [5,6,7]. Therefore, the development of novel membrane materials with improved selectivity and antifouling capabilities is necessary. The electrically conductive membrane (also named conductive membrane) represents a rapidly developing field and shows great potential in wastewater treatment [8,9,10,11].

With the assistance of an external electric field, the conductive membrane acts as a working electrode and filtration membrane, achieving the dual effects of physical separation of pollutants and electrochemical degradation simultaneously. Moreover, the clogging of membrane pores and the development of a cake layer can be efficiently prevented by electrochemical redox reactions, electrostatic repulsion, and produced gas scouring between electro-conductive membranes and contaminants, hence mitigating membrane fouling [12,13,14,15]. In electrochemical membrane bioreactors (EMBRs), conductive membranes are directly used as electrodes; they have gained much attention for excellent antifouling performance and space-saving characteristics. A series of approaches such as surface coating, chemical polymerization, and blending modification have been employed to fabricate or modify the conductive membranes that can serve as anodes or cathodes [16,17,18], facilitating in situ control of electrochemical reactions to achieve cost-effective membrane fouling control [19,20,21].

In recent years, electrically conductive polymers have attracted significant attention from researchers because of their capacity to perform reversible redox reactions, which result in modifications to conductivity, color, and volume during functional material fabrication [22]. Electrically conductive polymers have the potential to be used in many applications, including membrane separation [23], supercapacitors [24], electrochemical sensors [25], as well as mechanical actuators [26]. Polymers like polypyrrole, polyacetylene polyaniline, and polythiophene are ideal organic materials for tunable separation membranes, offering biocompatibility, cost-effectiveness, and good compatibility. Among electrically conductive polymers, polypyrrole is one of the most studied materials due to its excellent conductivity (with conductivity ranging from 0.1 to 7500 S/cm), simple preparation methods, low density, and wide application prospects [27,28]. Recent studies have shown that electrically conductive polymer membranes exhibit improved permeation performance, antifouling properties, and separation of charged pollutants. Benson et al. utilized an electrodeposition technique to integrate electrically conductive polyaniline onto nonwoven carbon nanotube (CNT) fabric [29]. The resulting high-strength conductive membranes demonstrated rapid ion adsorption and impressive specific capacitances, which render them ideal for low-energy desalination through capacitive deionization. Dudchenko et al. developed electrically conductive membranes (2500 S/m) composed of cross-linked polyvinylalcohol (PVA) and carboxylated multi-walled carbon nanotubes (PVA-CNT-COOH) using a sequential deposition and cross-linking method [30]. The effects of applied extern voltage on membrane fouling when filtering alginic acid and poly(ethylene) oxide solutions were studied, and the membrane fouling rate was decreased by 51% at −5 V compared with the control group. However, it remains a challenge to optimize the modification conditions to prepare high-quality conductive membranes with high conductivity, hydrophilicity, and porous structure [31] under the circumstance of increasing attention being paid to electrified wastewater treatment in nowadays.

In this study, the in situ oxidative polymerization method was employed to prepare polyvinylidene fluoride based conductive membranes (PVDF-CMs). To achieve high conductivity and hydrophilicity of the prepared PVDF-CMs, response surface methodology (RSM) was utilized to explore the relationship between the modification conditions and membrane properties including electrical resistance and water contact angle. The preparation conditions for the PVDF-CMs were optimized through multiple quadratic regression equations and variance analysis. The control PVDF membrane and PVDF-CMs were characterized using four-probe electrical resistance meter, Fourier transform infrared spectroscopy (FTIR), scanning electron microscopy with energy dispersive X-ray spectroscopy (SEM-EDX), static contact angle measurements, as well as an electrochemical workstation. To verify the antifouling performance of the PVDF-CM, batch filtration experiments were conducted using natural organic pollutants such as bovine serum albumin (BSA), sodium alginate (SA), and humic acid (HA) to analyze the filtration behaviors and antifouling properties. This study provided new insights into the preparation of cost-effective and highly conductive membranes for electrified wastewater treatment.

## 2. Materials and Methods

### 2.1. Materials

The supporting material for preparing conductive membrane is a commercial polyvinylidene fluoride (PVDF) membrane (Chongqing Jichuang Technology Co., Ltd., Chongqing, China), with a pore size of 0.45 μm. The pyrrole and 1-vinyl-3-butylimidazolium tetrafluoroborate (BVIMBF_4_) were purchased from Shanghai Macklin Biochemical Technology Co., Ltd., Shanghai, China, with the structural formula shown in Table 1. Hexahydrate ferric chloride (FeCl_3_·6H_2_O), ethanol (C_2_H_5_OH), sodium hydroxide (NaOH), hydrochloric acid (HCl), and sodium chloride (NaCl) were acquired from Tianjin Kemiou Chemical Reagent Co., Ltd., Tianjin, China. Bovine serum albumin (BSA, Biochemical reagent) and sodium alginate (SA, Chemically Pure) as well as humic acid (HA, Biological reagent) were obtained from China National Pharmaceutical Group Chemical Reagents Co., Ltd., Shanghai, China. Humic acid (HA, Biological reagent) was obtained from Shanghai Zhanyun Chemical Co., Ltd., Shanghai, China. The average molecular weights of BSA, SA, and HA are 68 kDa, 160 kDa, and 3.5 kDa, respectively.

### 2.2. Preparation and Characterization of Conductive Membrane

#### 2.2.1. Modification of the Conductive Membrane

In situ oxidative polymerization was employed to prepare conductive membranes [32]. The PVDF-based membrane was first soaked in pure water for more than 24 h, then removed and dried in the air. A certain concentration of pyrrole (with ethanol as the solvent) was prepared in 20 mL and thoroughly mixed with a specific amount of BVIMBF_4_. The pre-treated PVDF membrane was immersed in the prepared pyrrole solution for 30 min (flipping every 5 min), then transferred to the FeCl_3_·6H_2_O solution for a reaction lasting 30 min (conducted at low temperatures). The membrane surface was washed sequentially with 0.1 mol/L hydrochloric acid, ethanol, and pure water to remove any residuals. Finally, the modified membrane (namely PVDF-CM) was dried in air for 30 min.

Response surface methodology (RSM) is a prevalent tool that statistically observes the relationship (or response) between experimental indicators and various factors for process optimization [33]. As presented in Table 2, a three-factor and three-level Box-Behnken design (BBD) was conducted using Design-Expert 12 software (12.0.3.0), and a second-order RSM was utilized for regression fitting of the experiments to optimize modification conditions of the conductive membrane. The concentrations of pyrrole (A), BVIMBF_4_ (B), and FeCl_3_·6H_2_O (C) were selected as the response variables, with electrical resistance and water contact angle as the response values. The experiment data were fitted using a second-order polynomial equation, as shown in Equation (1), after conducting a total of 17 experiments.

(1) $$Y=b_{0}+\sum_{i=1}^{k}biXi+\sum_{i=1}^{k}{biiXi}^{2}+\sum_{i=1}^{k}\sum_{j>1}^{k}bijXiYj$$

where Y is the response value of the conductive membrane; b_0_ is the constant term; bi is the regression coefficient for linear effects; bii is the regression coefficient for quadratic effects; bij is the regression coefficient for interaction effects; and Xi is the coded experimental variable. The coefficient of determination R^2^ was used to evaluate the extent of interaction among experimental factors affecting the observed response values, with values ranging from 0 to 1 [34]. If the R^2^ value is close to 1, the model can predict the response better.

#### 2.2.2. Characterization of the Conductive Membrane

Scanning electron microscopy (SEM; Quanta650FEG, FEI, Hillsboro, OR, USA) was used to analyze the surface morphology of the PVDF membrane and PVDF-CM, and energy-dispersive X-ray spectroscopy (EDX; Quanta650FEG, FEI, Hillsboro, OR, USA) was employed to determine the elemental content. Fourier transform infrared spectroscopy (FTIR; Nicolet iS50, Thermo, Waltham, MA, USA) was utilized to characterize the functional groups of the PVDF membrane and the PVDF-CM. Four-probe electrical resistance meter (HPS2523, HAIERPA, Changzhou, China) was used to measure the electrical resistance of the conductive membranes, with each membrane measured more than 10 times and the average values reported as the final results. The static water contact angle of pure water on both the PVDF and the PVDF-CM was determined using a contact angle goniometer (SL200A, Kino, Shanghai, China), with each membrane measured three times, and the mean value was reported as the final result. A liquid zeta potential meter (ZS90, Malvern, Malvern City, Worcs, UK) was used to measure the zeta potential of model pollutants. An electrochemical workstation (PAR2273, Ametec, San Diego, CA, USA) was used to characterize the electrochemical impedance spectroscopy (EIS) and cyclic voltammetry (CV) curves of the conductive membrane, with an electrolyte of 0.1 mol/L NaCl solution, a reference electrode of Ag/AgCl, and a counter electrode of platinum electrode. EIS parameters were listed as follows: amplitude of 5 mV and frequency ranging from 0.01 to 100,000 Hz. CV parameters were shown below: a scan range of −2 to 2 V and a scan speed of 0.05 V/s for 4 cycles.

### 2.3. Anti-Fouling Performance of Conductive Membranes

For the pure water flux measurement, the pump speed was adjusted from low to high levels, and the transmembrane pressure (TMP) was recorded at different pump speeds. Pure water was filtered through the membrane for 4 min, followed by a 1 min pause. Permeate volume under each pump speed was measured three times with the average value used, and Equation (2) was employed to calculate the flux.

(2) $$J=\frac{V}{At}$$

where J is the water flux (LMH), V is the volume of the permeate for a certain measurement time (L), A is the membrane area (m^2^), and t is the measurement time (h).

**Figure 1:**
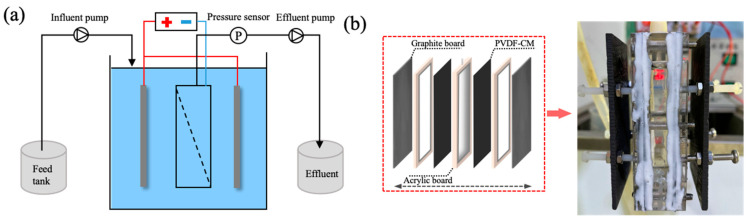
Schematic diagrams of the experimental setup (**a**); membrane module (**b**).

A membrane filtration device was used to assess the filtration performance of conductive membranes, as shown in Figure 1. Influent feeding and effluent extraction were achieved with the assistance of two peristaltic pumps. The TMP evolution during the operation was recorded based on an online pressure sensor. The effective filtration area of the flat-sheet PVDF-CM module is 40 cm^2^ in total. Conductive membranes were responsible for the cathodes and graphite plates were used as the anodes, with a distance of 1.5 cm between them. A DC power supply (eTM-305P, TONGMENG, Dongguan, China) was used to generate electrical potentials of 0 V, 0.5 V, 1.0 V, and 2.0 V for the experiment. BSA, SA, and HA were selected as the model pollutants at a concentration of 100 mg/L. The pH values of BSA and SA solutions prepared are in a range of 6.5 to 7.5. The preparation of HA solution involved stirring it magnetically for 24 h in a 0.01 mol/L NaOH solution, and then diluting it with a 0.01 mol/L HCl solution to reach pH 7. The solution temperature was maintained at 25 °C. In the batch filtration experiments, the initial flux was set to 36 LMH, effluent samples were taken every 20 min, and the concentrations of polysaccharides, proteins, and humic substances were analyzed using the anthrone-sulfuric acid method [35], the Folin–Lowry method [36], and UV_254_ absorbance measurement, respectively.

The permeability of the membranes was used to assess their filtration performance, which was calculated based on Equation (3):

Permeability = Flux/TMP(3)

where Flux and TMP is the flux (LMH) and transmembrane pressure (bar) in membrane filtration, with the unit of the permeability expressed as LMH/bar.

The rejection performance of the membranes for BSA, SA, and HA filtration was calculated using Equation (4):

R = (1 − c_b_/c_a_) × 100% (4)

where R is the rejection rate (%) and c_b_ and c_a_ are the concentrations of pollutants in the permeate and feed, respectively (mg/L).

## 3. Results and Discussion

### 3.1. Optimization of Conductive Membrane Preparation Methods

#### 3.1.1. Regression Model and Variance Analysis

The RSM was utilized to optimize experimental conditions, facilitating the selection of the best experimental scheme. A total of 17 designed experiments were conducted in no fixed order to reduce external influences on the experimental results. Table 3 summarizes the 17 experimental schemes and the response values of the conductive membrane.

To obtain an accurate and effective mathematical model, multi-variable nonlinear fitting analysis was performed based on the results from Table 3. The variance analysis for the regression model is listed in Table S1. Additionally, a multiple regression fitting analysis was conducted to establish second-order polynomial regression equations for the target functions of electrical resistance (Y_1_) and water contact angle (Y_2_) in relation to the variables, namely the concentrations of pyrrole (A), BVIMBF_4_ (B), and FeCl_3_·6H_2_O (C), as shown in Equations (5) and (6).

Y1 = 431.47 − 214.13A − + 263.56B − 1517.53C − 19.19AB + 257.07AC − 163.88BC + 96.40A^2^ + 283.71B^2^ + 1236.42C^2^
(R_1_^2^ = 0.9907) (5)

**Figure 2:**
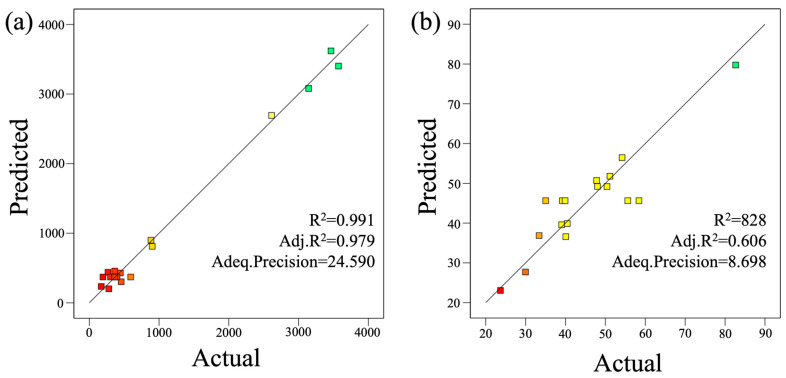
Actual values from experiments and RSM-predicted values for (**a**) electrical resistance and (**b**) water contact angle. (note: The color changes in the image represent variations in data values across different areas. Specifically, a gradient from green to yellow to red indicates a decrease in values, while the speed of the color transition reflects changes in slope).

Y_2_ = 38.97 − 6.68A − 24.44B − 7.08C + 1.46AB + 8.01AC + 0.43BC + 7.71A^2^ − 19.43B^2^ + 3.88C^2^
(R_2_^2^ = 0.8276) (6)

where Y_1_ is electrical resistance (Ω/sq), Y_2_ is the water contact angle (°), and A, B, and C are the encoded forms of the variables, displayed in singular form to show the influence of each factor. The interaction terms of AB, AC, and BC represent the interactions between the variables, while A^2^, B^2^, and C^2^ denote the squared terms of each factor [37]. The corrected R^2^ values for the average values predicted by Equations (5) and (6) are 0.98 and 0.61, respectively, whereas the R^2^ values of the models are 0.99 and 0.83, as shown in Figure 2. The signal-to-noise ratios for Y_1_ and Y_2_ models are 24.59 and 8.70, respectively, both greater than 4, indicating that the model has accurate predictive capabilities for the experimental data [38,39].

A smaller probability value (*p* value) can indicate the significance of the corresponding coefficients [40]. From Table S1, the ANOVA shows that the *p*-values for both Y_1_ and Y_2_ models are less than 0.05, indicating that the RSM model analysis is statistically significant. The lack of fit for Y_1_ and Y_2_ is 0.22 and 0.91, respectively, both greater than 0.05 (*p* > 0.05), indicating that the model can precisely replicate the modification conditions of the conductive membrane [41].

#### 3.1.2. Data Analysis and Optimization

**Figure 3:**
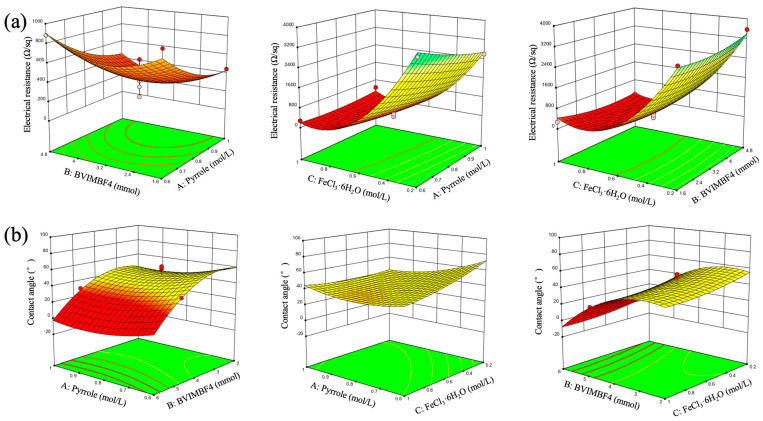
RSM diagrams of response values optimized for the PVDF-CM: (**a**) electrical resistance, (**b**) water contact angle. (note: The color changes in the image represent variations in data values across different areas. Specifically, a gradient from green to yellow to red indicates a decrease in values, while the speed of the color transition reflects changes in slope).

Based on the ANOVA table and model selection, three-dimensional response surface plots were drawn to analyze the interaction effects of pyrrole, BVIMBF_4_, and FeCl_3_·6H_2_O on the electrical resistance and water contact angle of the prepared conductive membrane, as shown in Figure 3. The electrical resistance of the conductive membrane initially decreases and then increases with the addition of BVIMBF_4_, displaying a parabolic response surface, as shown in Figure 3a. The lowest electrical resistance of 365 Ω/sq is achieved when the BVIMBF_4_ concentration is 3.2 mmol because the BVIMBF_4_, which possesses excellent ionic characteristics, provides some proton sources as a green reaction medium with their anions doped into the molecular chains [42]. The larger molecular weight and volume favor the stretching of the polypyrrole molecular chains, thereby improving conductivity. However, excessive addition of BVIMBF_4_, due to its viscosity, can hinder the diffusion of the pyrrole, leading to reduced polymerization and increased electrical resistance. When the amount of BVIMBF_4_ is held constant, the electrical resistance exhibits a similar pattern of first declining and then increasing with the addition of pyrrole, with the lowest electrical resistance occurring at a pyrrole concentration of 0.81 mol/L. This is attributed to the increased content of polypyrrole on the membrane surface, enhancing conductivity. However, further increase in pyrrole concentration does not significantly improve conductivity. The electrical resistance also exhibits a tendency of lowering and subsequently increasing with variations in FeCl_3_·6H_2_O concentration under constant concentrations of pyrrole and BVIMBF_4_, reaching a minimum electrical resistance of 196 Ω/sq near 0.7 mol/L.

From Figure 3b, it can be observed that the water contact angle of the conductive membrane decreases overall with the increase in BVIMBF_4_. A higher BVIMBF_4_ content results in a greater quantity of BVIMBF_4_ doping into the pyrrole, which improves the membranes’ hydrophilicity and lowers the water contact angle. The effects of pyrrole and FeCl_3_·6H_2_O concentrations on the water contact angle seem to be minimal, resulting in a relatively flat response surface. This is due to a layer of polypyrrole adhering to the membrane surface, and when the concentration exceeds a certain level, it has little impact on the hydrophilicity of the membrane.

Using the Design-Expert software (12.0.3.0) for data processing and analysis, the significance of the three factors on electrical resistance and water contact angle as well as the application of the conductive membrane in EMBR was evaluated, resulting in the optimal design for preparing conductive membranes, which maintains high conductivity while achieving high hydrophilicity. The specific results are shown in Figure S1.

According to the optimized predictions, the best conditions for preparing the conductive membrane are pyrrole, BVIMBF_4_, and FeCl_3_·6H_2_O concentrations of 0.84 mol/L, 0.87 mol/L, and 4.84 mmol, which yield an electrical resistance of 73.17 Ω/sq and a water contact angle of 23.08°. To facilitate experimental manipulation, pyrrole, BVIMBF_4_, and FeCl_3_·6H_2_O concentrations of 0.90 mol/L, 4.80 mmol, and 0.80 mol/L were selected. To verify that the optimal values of the model are accurate, three sets of validation experiments were conducted under these experimental conditions, with results listed in Table 4. In the actual experiments, the electrical resistance and water contact angle of the conductive membrane were 93 Ω/sq and 31 °, respectively, which are in good accord with the theoretical predictions of the model. Therefore, it can be concluded that the best conditions obtained through response surface optimization are highly reliable and have practical reference value.

### 3.2. Performance of the Conductive Membranes

#### 3.2.1. Permeability and Physicochemical Properties

**Figure 4:**
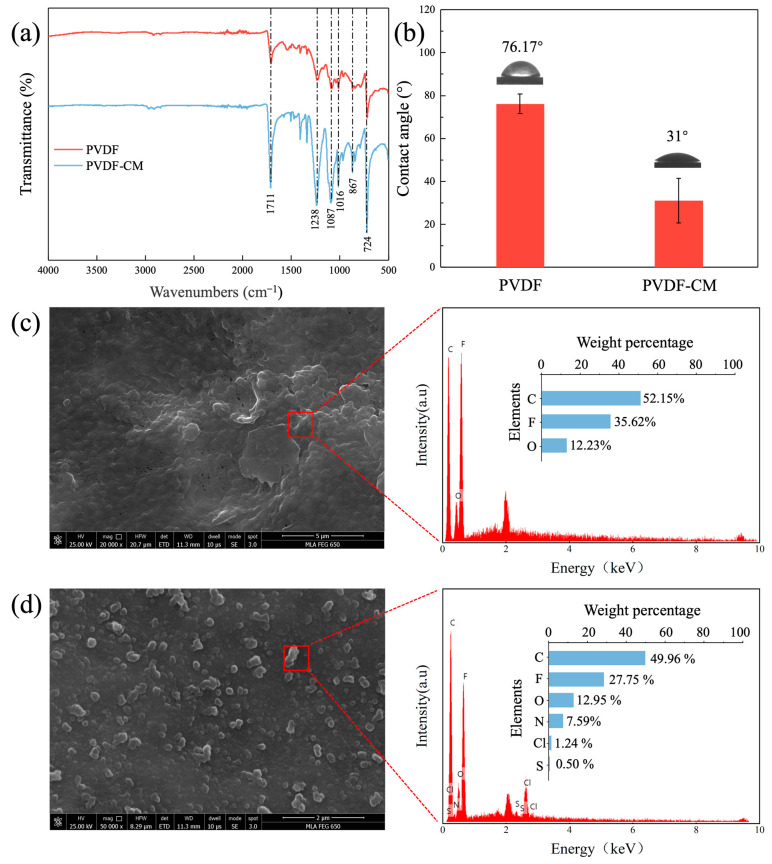
Structural and chemical composition analysis of PVDF and PVDF-CM. (**a**) FTIR, (**b**) water contact angle; SEM-EDX analysis of the PVDF (**c**) and PVDF-CM (**d**).

The PVDF-CM prepared by in situ oxidative polymerization showed a notable visual change. The surface color of the PVDF-CM changed from white to a very deep and uniform black, indicating that polypyrrole has polymerized on the surface of PVDF membrane. Figure 4a presents FTIR analysis of the PVDF membrane and PVDF-CM. Compared to the PVDF membrane, PVDF-CM exhibits an absorption peak at 1711 cm^−1^ corresponding to the N-H bond on the pyrrole ring, and a peak at 1238 cm^−1^ for the stretching vibrations of C-N in the pyrrole ring. The range of 900 to 650 cm^−1^ shows out-of-plane bending vibrations of the N-H bond. The absorption peak for the C=N bond of the imidazole ring in the BVIMBF_4_ appears between 1711 and 1238 cm^−1^. Most of the absorption peaks between 1000 and 500 cm^−1^ are due to various in-plane bending vibrations of C-H in the BVIMBF_4_, while the peak at 1016 cm^−1^ corresponds to the stretching vibrations of the B-F bond.

Figure 4b illustrates the water contact angles of the PVDF membrane and PVDF-CM, examining the hydrophilicity of the membranes. When the water contact angle is less than 90°, it means that the material is hydrophilic, with a smaller contact angle reflecting greater hydrophilicity. The water contact angle of the PVDF membrane is 76.17°, while that of the PVDF-CM is 31°. Polymerization of polypyrrole on the membrane surface has been shown to enhance the hydrophilicity of PVDF-CM. Current understanding of the hydrophilicity of membrane bioreactor (MBR) membranes suggested that membranes with better hydrophilicity exhibit better anti-fouling capability [43].

**Figure 5:**
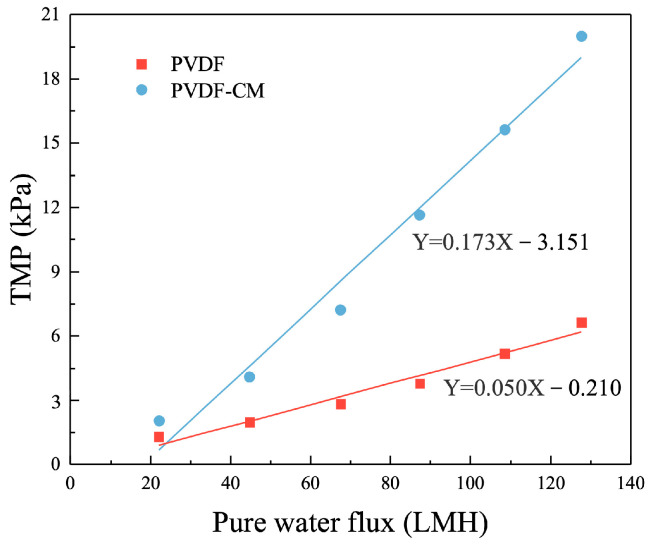
The filtration performance of PVDF and PVDF-CM in pure water.

To investigate the formation and adhesion of the polymers on membrane surface, a microscopic comparative analysis of the surface morphology of PVDF membrane and PVDF-CM was conducted. Figure 4c shows that the PVDF membrane has a relatively rough and uneven surface, with a fairly uniform pore structure. In contrast, Figure 4d shows that PVDF-CM exhibits a relatively uniform microstructure, indicating that the addition of polypyrrole has influenced the membrane morphology, leading to the formation of coral-like clusters on the membrane surface with some pore coverage. The pure water flux tests (Figure 5) showing a decrease in membrane permeability confirm this finding. The presence of C, F, and O elements was found in both PVDF membrane and PVDF-CM after analyzing the element contents using EDX. However, due to the deposition and adhesion of polypyrrole and minor residuals on the membrane surface, elements N and Cl were found to be increased in PVDF-CM, while the reduction in C and F contents was attributed to the deposition of polypyrrole.

Figure 5 shows the relationship between TMP and pure water flux for PVDF membrane and PVDF-CM. Within a certain flux range, the permeability of pristine PVDF membrane is between 1703.02 and 2383.67 LMH/bar, with a fitted average value of 2118.13 LMH/bar. In contrast, the permeability of the PVDF-CM ranges from 591 to 1088.62 LMH/bar, with a fitted mean value of 832.29 LMH/bar. It indicates that the permeability decreases by 60.7% after membrane modification. This reduction is primarily due to the dense layer of polypyrrole formed on the PVDF membrane surface, which may partially block the membrane pores and lead to decreased permeability [44,45].

#### 3.2.2. Electrochemical Performance of Conductive Membranes

**Figure 6:**
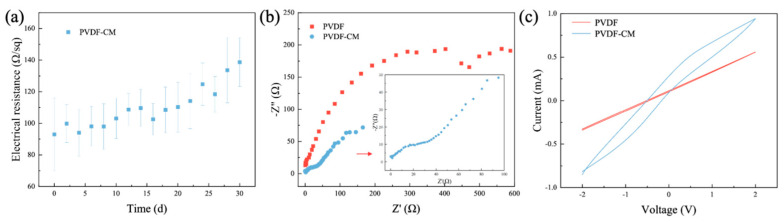
The conductivity of PVDF-CM changes with time in air (**a**); EIS (**b**) and CV (**c**) of PVDF membrane and PVDF-CM.

Figure 6a shows the change in electrical resistance of the PVDF-CM in air over 30 d. The initial electrical resistance was 93 Ω/sq, and with prolonged exposure time the electrical resistance gradually increased, indicating a decrease in conductivity. After 30 days, the electrical resistance reached 138.7 Ω/sq, but the conductive membrane still exhibited good conductivity compared with previous studies [32,46,47].

Figure 6b,c display the EIS (Nyquist plots) and CV curves of the PVDF membrane and PVDF-CM, respectively. The impedance of the PVDF membrane shows a semicircular arc in the low-frequency range (Figure 6b), demonstrating that electron transfer is hindered due to the non-conductive nature of the PVDF material. On the contrary, the impedance of the PVDF-CM approaches a linear trend in the low-frequency region, indicating a significant enhancement in the electron transfer rate at the membrane interface due to the attachment of a conductive polypyrrole layer on the membrane surface, which facilitates electron transfer [48].

Figure 6c illustrates the CV curves of the membranes, showcasing the redox characteristics before and after conductive modification. Within the scanning range, the CV curve of PVDF membrane appear flattened and nearly closed, with a current range of -0.4 to 0.6 mA at the same voltage, indicating stable redox characteristics of the PVDF membrane. On the other hand, the CV curve of the PVDF-CM is broader compared to that of the PVDF membrane, indicating that no significant electrochemical reactions occurred within the scanning range of the conductive material in the membrane. This suggests that the PVDF-CM has stable oxidative current characteristics, with a current range of −0.9 to 1.0 mA, demonstrating a higher current density than the PVDF membrane, which is in line with EIS results [49]. Therefore, the PVDF-CM prepared with polypyrrole as the conductive layer demonstrated good stability, conductivity, and relatively stable redox characteristics.

### 3.3. Anti-Fouling Performance of Conductive Membranes

**Figure 7:**
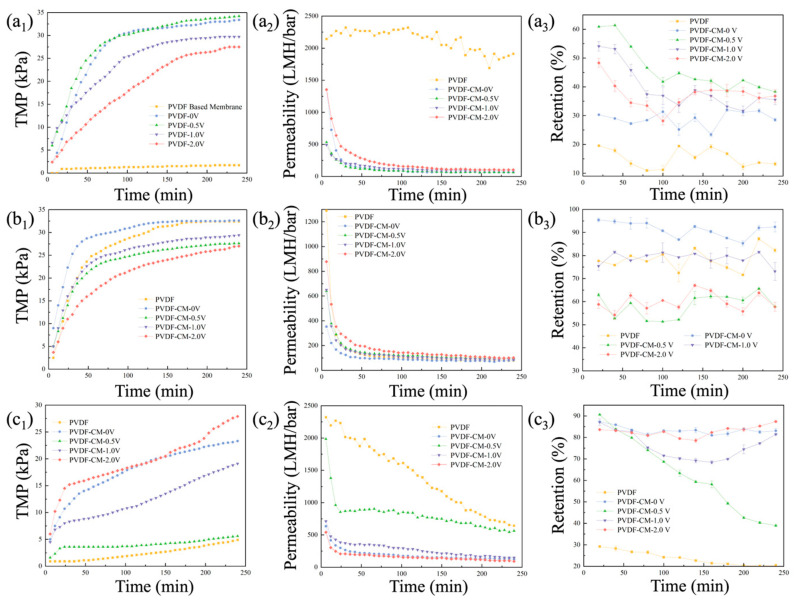
The variations of TMP, permeability, and pollutants retention with filtration time using PVDF membrane and PVDF-CM under varied external voltages filtering model pollutants: (**a**) BSA solution: TMP (**a_1_**); Permeability (**a_2_**); Retention (**a_3_**); (**b**) SA solution: TMP (**b_1_**); Permeability (**b_2_**); Retention (**b_3_**); and (**c**) HA solution: TMP (**c_1_**); Permeability (**c_2_**); Retention (**c_3_**).

The experimental results of the PVDF membrane and PVDF-CM in BSA filtration are shown in Figure 7a at a constant flux filtration mode (36 LMH). The changes in TMP and membrane permeability over filtration time (4 h) are illustrated in Figure 7a. During the 4 h filtration, the TMP of the PVDF membrane remained stable at around 1.7 kPa. When applying external voltages of 0V, 0.5 V, 1.0 V, and 2.0 V, the TMP growth rates for PVDF-CM were 8.35, 8.55, 7.43, and 6.87 kPa/h, respectively. The fouling rate of the modified membrane increased mainly due to the attachment of a polypyrrole layer, which partially blocked the membrane pores and reduced permeability. After applying a 2.0 V electric field, the fouling rate of the PVDF-CM was the lowest, decreasing by 17.72% compared to 0 V.

**Figure 8:**
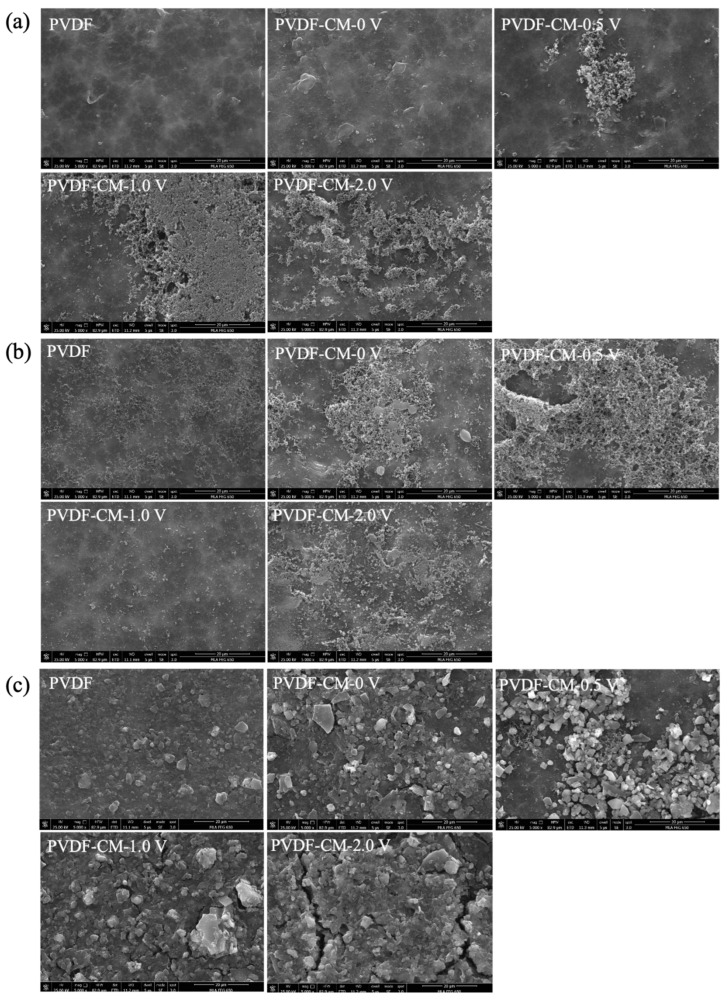
SEM images of the PVDF membrane and PVDF-CM fouled at different voltages by BSA (**a**), SA (**b**) and HA (**c**).

Figure 8a provides SEM images supporting this observation, showing that the surface of the PVDF membrane had fewer attached foulants, while the PVDF-CM at 0 V had a denser accumulation of contaminants. This phenomenon is likely caused by the changes in surface morphology, structure, and porosity before and after membrane modification. After applying an external electric field, the contaminants are more loosely attached and less dense on the membrane surface of the PVDF-CM, primarily due to the negatively charged protein molecules, which enhanced the electrostatic repulsive force between the membrane and the contaminants as the voltage increases within a certain range. This change in movement direction and material morphology effectively reduced the accumulation of contaminants on the membrane surface and within its pores [50]. However, at 0.5 V, the fouling rate slightly increased, possibly because of the accumulation of contaminants around the membrane at low potential. Previous research showed that contaminants are typically negatively charged, and applying a negative potential on the conductive membrane is beneficial for controlling membrane fouling [51,52]. However, in this study, the conductive membrane acted as a cathode, and the pollution worsened when applying 0.5 V, challenging the conventional understanding that negative potentials alleviate membrane fouling. Similar observations were noted by Ying et al. in an AnEMBR study, showing that applying 0.5 V to a conductive ceramic membrane also led to increased fouling [10].

The retention performance of the membranes is illustrated in Figure 7a_3_. PVDF exhibits almost no retention effect on proteins, with the retention rate of PVDF-CM at 0 V being relatively low (approximately 25.7%). However, with the application of external voltage, the initial retention rate during filtration was higher, subsequently declining but still remaining at a relatively high level. The average retention rates of proteins by the PVDF-CM at voltages of 0 V, 0.5 V, 1.0 V, and 2.0 V were 28.90%, 46.05%, 40.72%, and 36.01%, respectively. The membrane surface with a dense fouling layer effectively retains pollutants, leading to its highest retention rate at 0.5 V.

The experimental results of the PVDF membrane and PVDF-CM in SA filtration are shown in Figure 7b. At 0 V, PVDF-CM showed a rapid increase in TMP during the first 2 h of filtration, but after 4 h, the TMP became similar to that of the PVDF membrane, with a membrane fouling rate of 8.15 kPa/h. After applying external voltage, the TMP growth rates for the PVDF-CM at 0.5 V, 1.0 V, and 2.0 V were 6.90, 7.35, and 6.75 kPa/h, respectively, low compared to both PVDF and PVDF-CM at 0 V. As the voltage increased, membrane fouling rate declined more significantly, primarily because the pollutants carry a negative charge. The increased voltage enhances the electrostatic repulsive force between the membrane and the pollutants. When applying 2.0 V, the membrane fouling rate decreased by 17.18% compared to 0 V. From the SEM images in Figure 8b, it can be observed that the fouling layer with adhered pollutants on the surface of PVDF membrane and PVDF-CM at 0 V was denser, while the accumulated pollutants were obviously reduced after applying external voltage. The retention performance of the membranes is shown in Figure 7b_3_. The average retention rate of PVDF-CM at 0 V is 90.56%, while that of PVDF membrane is 78.50%. However, after applying external voltage, the retention rates of PVDF-CM for SA decreased. The average retention rates at external voltages of 0.5 V, 1.0 V, and 2.0 V were 57.99%, 78.52%, and 60.63%, respectively. The decrease in the retention effect after applying an external electric field may be attributed to the direct oxidation on the anode surface by hydroxyl radicals generated through physical adsorption or active oxygen through chemical adsorption, as well as the indirect oxidation by oxidants produced at the anode that diffuse into the solution [53]. This process can oxidize large molecular SA into smaller molecules (or degradation products), making it easier for them to transfer within the well-mixed solution and to pass through the membrane pores, resulting in a higher concentration of polysaccharides in the permeate.

The experimental results of the PVDF membrane and PVDF-CM in HA filtration are presented in Figure 7c. The TMP growth rate for PVDF membrane is 1.23 kPa/h, while the TMP growth rates for PVDF-CM at external voltages of 0 V, 0.5 V, 1.0 V, and 2.0 V are 5.83, 1.40, 4.85, and 6.98 kPa/h, respectively. Similar to BSA filtration, the PVDF membrane has fewer contaminants attached to its surface, whereas the PVDF-CM membrane has a denser layer of contaminants. This is primarily due to changes in the surface morphology, structure, and porosity of the modified membrane. At 0.5 V, the membrane fouling rate is similar to that of PVDF membrane, which represents a reduction of 76% compared to 0 V. However, at 2.0 V, the membrane fouling rate increases. It is reported that HA molecules easily undergo dipolarization under a higher electric field, causing the distribution of positive and negative charges within the molecules to become polarized at both ends. This leads to aggregation and a stronger affinity between the molecules and the membrane [54]. The SEM images in Figure 8c help explain this phenomenon; at 0.5 V, the membrane surface has a fouling layer that is looser than at 0 V, while the fouling layer at 2.0 V is denser. The membrane retention performance is shown in Figure 7c_3_. The average retention rate of PVDF membrane is 23.51%. The average retention rates of PVDF-CM at external voltages of 0 − 2.0 V are 83.14%, 62.80%, 78.65%, and 84.21%, respectively. After conductive modification, the PVDF-CM exhibits improved retention performance for HA pollutants, although retention performance decreases with the application of voltage. As the external voltage increases, the retention performance of the membrane gradually improves.

### 3.4. Implication of This Study

In this study, the preparation of the PVDF-CM was accomplished via an in situ oxidative polymerization approach, achieving both good conductivity (93 Ω/sq) and hydrophilicity (water contact angle of 31°) under optimized electro-conductive modification conditions. PVDF-CM demonstrates excellent anti-fouling and retention capabilities assisted by a low-voltage electric field. Moreover, the specific concentrations of pyrrole, BVIMBF_4_, and FeCl_3_·6H_2_O used during the synthesis highlight the importance of material composition in the performance of conductive polymers. This may lead to further optimization of the desired electrochemical and physical properties of the materials, providing a reference for further exploration of the synthesis and applications of conductive polymers.

It is worth noting that the modified conductive membrane exhibits a phenomenon of “permeability–selectivity trade-off”, where the retention performance of the conductive membrane has improved, but at the cost of reducing permeation performance substantially. In future research, it is considered to add pore-forming agents when preparing the conductive membrane aiming to increase membrane porosity and pore size and enhance membrane permeability [55,56]. In this study, the antifouling properties of the cathodic conductive membrane were explored, while it is of great interest to apply the conductive membrane as an anode to verify the anodic oxidation characteristics in mitigating membrane fouling and prolonging filtration duration [13], thereby further reducing the costs associated with membrane chemical cleaning practice.

## 4. Conclusions

In this study, an in situ oxidative polymerization approach was adopted to prepare PVDF-CM, and the RSM method was applied for optimizing the modification conditions. The prepared PVDF-CM exhibited favorable properties in terms of conductivity, permeability, stability, electrochemical characteristics, as well as anti-fouling performance. The following are the main conclusions:

(1) After optimization, the best conditions for preparing PVDF-CM were pyrrole concentration of 0.9 mol/L, BVIMBF_4_ concentration of 4.80 mmol, and FeCl_3_·6H_2_O concentration of 0.80 mol/L. The optimized PVDF-CM exhibited an electrical resistance of 93 Ω/sq and a water contact angle of 31°.
(2) The permeability of the control PVDF membrane was 2118.13 LMH/bar, while the permeability of PVDF-CM decreased to 832.29 LMH/bar, indicating a substantial reduction in permeability after modification.
(3) During batch filtration experiments, the application of an electric field at 2 V significantly reduced membrane fouling rates for BSA and SA, with a reduction of 17.72% and 17.18%, respectively. For HA, the membrane fouling rate decreased by 72.6% at 0.5 V.

In the future, the trade-off between permeability and selectivity of conductive membranes needs to be further investigated, and incorporating porogenic materials while ensuring that membrane conductivity and stability remain high can be a promising strategy.

## Figures and Tables

**Table 1:** The main chemicals used in this work.

Name	Molecular Formula	Structural Formula
Pyrrole	C_4_H_5_N	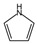
1-vinyl-3-butylimidazolium tetrafluoroborate	C_9_H_5_BF_4_N_2_	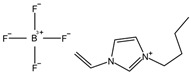
Polypyrrole	(C_4_H_5_N)_n_	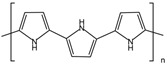

**Table 2:** Three-variable and three-level design using response surface methodology.

Factors	Parameters	Units	Code		
			−1	0	1
A	Pyrrole	mol/L	0.6	0.8	1.0
B	BVIMBF_4_	mmol	1.6	3.2	4.8
C	FeCl_3_·6H_2_O	mol/L	0.2	0.6	1.0

**Table 3:** Experimental design by the RSM-BBD and the obtained results.

Operating Factors				Results	
Run	A: Pyrrole (mol/L)	B: BVIMBF_4_ (mmol/L)	C: FeCl_3_·6H_2_O (mol/L)	Electrical Resistance (Ω/sq)	Water Contact Angle (°)
1	0.8	3.2	0.6	594.07	55.61
2	0.8	3.2	0.6	397.60	58.42
3	0.6	3.2	0.2	3464.67	82.66
4	1	4.8	0.6	365.40	29.97
5	0.8	3.2	0.6	304.00	35.03
6	1	3.2	1	458.00	47.80
7	0.6	4.8	0.6	884.73	40.45
8	0.8	3.2	0.6	196.40	39.22
9	0.6	3.2	1	281.07	48.00
10	1	3.2	0.2	2613.33	50.40
11	0.6	1.6	0.6	902.73	54.18
12	1	1.6	0.6	444.80	39.02
13	0.8	3.2	0.6	365.13	39.90
14	0.8	4.8	1	172.00	23.70
15	0.8	1.6	0.2	3144.67	51.14
16	0.8	4.8	0.2	3572.67	33.39
17	0.8	1.6	1	268.40	40.48

**Table 4:** A comparison of the optimal conditions for both models and experiments.

Parameter	Predicted Optimum Value	Experimental Optimum Value
Electrical resistance (Ω/sq)	73.17	93
Water contact angle (°)	23.08	31
Pyrrole (mol/L)	0.87	0.9
BVIMBF_4_ (mmol)	4.84	4.8
FeCl_3_·6H_2_O (mol/L)	0.84	0.8

## Data Availability

The original contributions presented in this study are included in the article/Supplementary Material. Further inquiries can be directed to the corresponding author.

## Supplementary Materials

The following supporting information can be downloaded at: https://www.mdpi.com/article/10.3390/membranes15010001/s1, Figure S1: The optimal reaction conditions; Figure S2: The zeta potential of model pollutants.; Table S1: ANOVA for the response surface of electrical resistance and contact angle.
