# Supplementary material for: Electro-Conductive Modification of Polyvinylidene Fluoride Membrane for Electrified Wastewater Treatment: Optimization and Antifouling Performance
Source: Membranes (Basel). 2024 Dec 24;15(1):1. doi: 10.3390/membranes15010001 (PMC11767159; doi:10.3390/membranes15010001)
Supplement: Supplementary file 1 [file membranes-15-00001-s001.zip › membranes-3357564-supplementary.pdf]

Supporting Information

Electro-conductive modification of PVDF membrane for electrified wastewater treatment: optimization and antifouling performance

Jinzhuo Shi[a,b], Yisong Hua[,b,*], Songhua Li[a,b], Wenqian Xiao[a,b], Yuan Yang[a,b], Jiayuan Ji[c,*]

a. Shaanxi Key Laboratory of Environmental Engineering, Xi’an University of Architecture and Technology, Xi’an 710055, China;

b. Key Laboratory of Northwest Water Resource, Environment and Ecology < Ministry of Education >, Xi’an University of Architecture and Technology, Xi’an 710055, China)

c. Institute for Future Initiatives, The University of Tokyo, 7-3-1 Hongo, Bunkyo-ku, Tokyo 113-8654, Japan

* Corresponding author

Name: A. Prof. Yisong Hu

Tel.: +8602982205652

E-mail: huyisong@xauat.edu.cn

Name: Dr. Jiayuan Ji

E-mail: kikaen@ifi.u-tokyo.ac.jp

Number of pages: 3

Number of figures: 2

Number of tables: 1

Table S1
ANOVA for the response surface of electrical resistance and contact angle.

Source	Electrical resistance (Ω/sq)					Water contact angle (°)				
	Sum of Squares	df	Mean Square	F-value	P-value	Sum of Squares	df	Mean Square	F-value	P-value
Model	2.44E+07	9	2.71E+06	82.44	＜0.0001	2426.57	9	269.62	3.73	0.0481
A	2.45E+05	1	2.45E+05	7.45	0.0294	237.99	1	237.99	3.30	0.1123
B	1.23E+05	1	1.23E+05	3.73	0.0946	1054.41	1	1054.41	14.60	0.0065
C	1.23E+07	1	1.23E+07	373.92	＜0.0001	267.15	1	267.15	3.70	0.0958
AB	942.49	1	942.49	0.0287	0.8703	5.48	1	5.48	0.0758	0.7910
AC	2.64E+05	1	2.64E+05	8.05	0.0252	256.64	1	256.64	3.55	0.1014
BC	68748.84	1	68748.84	2.09	0.1912	0.47	1	0.47	0.0065	0.9380
A^2	39131.30	1	39131.30	1.19	0.3112	250.02	1	250.02	3.46	0.1051
B^2	1.39E+05	1	1.39E+05	4.23	0.0789	651.25	1	651.25	9.02	0.0198
C^2	6.44E+06	1	6.44E+06	195.96	＜0.0001	63.33	1	63.33	0.88	0.3802
Residual	2.30E+05	7	32846.88			505.41	7	72.20		
Lack-of-Fit	1.45E+05	3	48150.91	2.25	0.2244	55.94	3	18.65	0.1659	0.9141
Pure Error	85475.43	4	21368.86			449.47	4	112.37		
Cor Total	2.46E+07	16				2931.97	16			

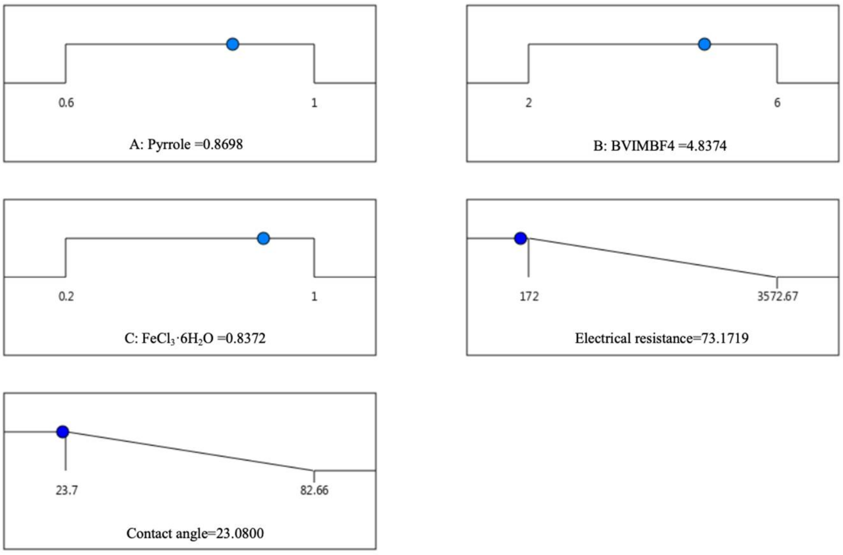

Fig. S1. The optimal reaction conditions.

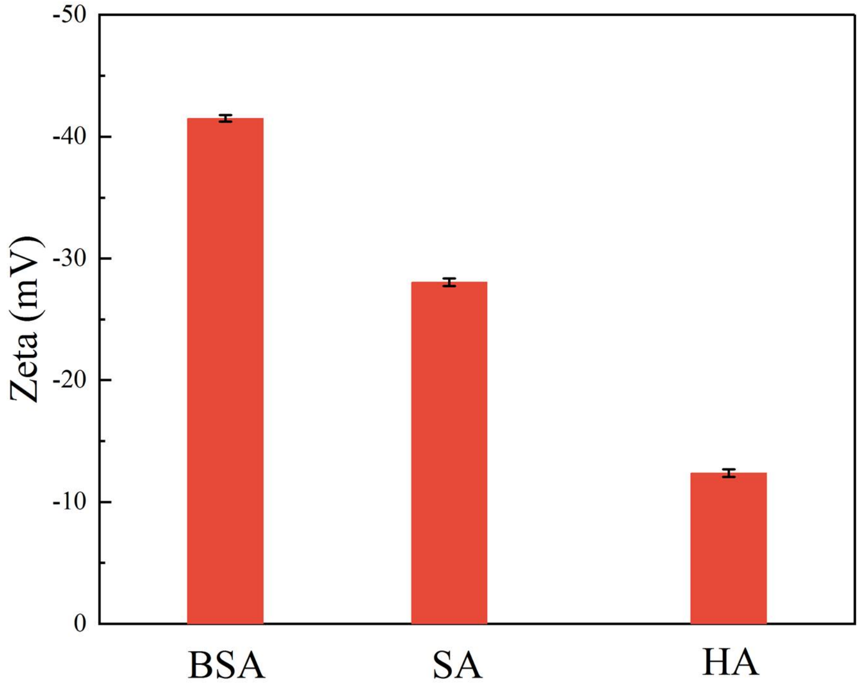

Fig. S2. The zeta potential of model pollutants.
